# Influences on pathologic complete response in breast cancer patients after neoadjuvant chemotherapy

**DOI:** 10.1007/s00404-021-06018-6

**Published:** 2021-03-10

**Authors:** Carolin Müller, Gilda Schmidt, Ingolf Juhasz-Böss, Lisa Jung, Sarah Huwer, Erich-Franz Solomayer, Stephanie Juhasz-Böss

**Affiliations:** 1 Department of Gynecology, Obstetrics and Reproductive Medicine, Saarland University Medical Center, Kirrbergerstraße 100, 66424 Homburg/Saar, Germany; 2 Department of Gynecology, Obstetrics and Reproductive Medicine, University Medical Center Freiburg, Freiburg, Germany

**Keywords:** Breast cancer, Neo-adjuvant therapy, Pathologic complete response, Clinical trials

## Abstract

**Purpose:**

Pathologic complete response is associated with longer disease-free survival and better overall survival after neoadjuvant chemotherapy in breast cancer patients. We, therefore, evaluated factors influencing pathologic complete response.

**Methods:**

Patients receiving neoadjuvant chemotherapy from 2015 to 2018 at the Saarland University Hospital were included. Patients’ age, tumor stage, tumor biology, genetic mutation, recurrent cancer, discontinuation of chemotherapy, and participation in clinical trials were extracted from electronic medical records. Binary logistic regression was performed to evaluate the influence of these factors on pathologic complete response.

**Results:**

Data of 183 patients were included. The median patient’s age was 54 years (22–78). The median interval between diagnosis and onset of chemotherapy was 28 days (14–91); between end of chemotherapy and surgery 28 days (9–57). Sixty-two patients (34%) participated in clinical trials for chemotherapy. A total of 86 patients (47%) achieved pathologic complete response. Patient’s age, genetic mutation, recurrent cancers, or discontinuation of chemotherapy (due to side effects) and time intervals (between diagnosis and onset of chemotherapy, as well as between end of chemotherapy and surgery) did not influence pathologic complete response. Patients with high Ki67, high grading, Her2 positive tumors, as well as patients participating in clinical trials for chemotherapy had a higher chance of having pathologic complete response. Patients with Luminal B tumors had a lower chance for pathologic complete response.

**Conclusion:**

Particularly patients with high risk cancer and patients, participating in clinical trials benefit most from chemotherapy. Therefore, breast cancer patients can be encouraged to participate in clinical trials for chemotherapy.

## Introduction

Neoadjuvant chemotherapy (NACT) has changed its role for only inoperable and locally advanced breast cancer to a treatment used in early stages of breast cancer [1]. Particularly patients at high risk, like patients with Her2 positive and triple-negative breast cancer benefit from receiving NACT [2, 3]. Pathological complete response (pCR) is associated with a better outcome, meaning that patients having pCR have longer disease-free survival and better overall survival [5].

The definition of pCR varies in individual studies (ypT0/ypTis/ypN0). However, it could be shown that no residual invasive cancer in the breast and axilla is associated with better outcomes compared with no residual cancer in the breast alone [4]. Von Minckwitz et al. even detected a longer disease-free survival in patients with no invasive and no in situ carcinoma in the breast and axilla [5]. Also, tumor subtypes influence the achievement of pCR and thus a better outcome. For example, LeVasseur et al. detected that patients with triple negative tumors had longer relapse-free-survival and breast cancer-specific survival when achieving pCR after NACT [6].

As pCR plays an important role in the outcome of breast cancer, several studies already evaluated possible influencing factors, like age of the patients [7, 8], tumor biology [9] or genetic mutation [10]. The majority of the cited studies analyzed data that was previously collected as part of NACT trials. But patients who are included in clinical studies are mostly a selected collective due to in- and exclusion criteria with fewer previous illnesses, or of a certain subtype or tumor size. For this reason, we wanted to analyze possible influencing factors on pCR in “real-world-data”.

Although, some factors such as age, tumor biology or genetic mutation cannot be influenced directly, it is still important to identify possible risk factors to maybe establish further follow-up therapies or intensified aftercare. We, therefore, determined various clinical factors from electronic patient records and evaluated their influence on the probability of pCR. Parts of the registry were used for two other analyzes before [11, 12]. We could show that especially inpatient and outpatient presentations of the patients delayed therapy onset of NACT and surgery [11, 12].

## Patients and methods

### Data collection

All patients receiving NACT due to newly diagnosed breast cancer between 2015 to 2018 at the Department of Gynecology, Saarland University Medical Center, were included in the study. The primary endpoint was pCR after NACT. pCR was defined as no residual tumor in the breast and axilla (ypT0, N0). It includes no residual Tis (Carcinoma in situ). Date of diagnosis was defined as the date of core biopsy. Patients’ age, tumor stage, eventual multi-centricity, and tumor biology were recorded. It was distinguished whether the patients received NACT as standard therapy or in a clinical trial. Mainly GAIN II [13], Gepar Octo [14], and Gepar X [15] were addressed from 2015 to 2018 in the Saarland University Medical Center as clinical trials. The time interval between diagnosis and onset of chemotherapy, as well as between end of chemotherapy and surgery, was recorded. The type of operation and duration of hospitalization were documented. Patients who had an indication for BRCA testing according to the guidelines of the German AGO Mamma and the German Consortium for Hereditary Breast and Ovarian Cancer (GC-HBOC) were tested for a BRCA mutation. Their recommendations are based on an analysis of 21,401 families [16]. It was registered whether patients had a genetic mutation (BRCA) or recurrent cancer. Recurrent cancer was defined as local recurrence of breast cancer. Patients with local recurrence were included in the study regardless of their previous therapy. If patients discontinued chemotherapy due to therapy complications or disease progress, it was recorded as well.

### Data management and statistics

Patients’ data were reviewed in the hospital’s digital documentation system (SAP, Walldorf, Germany). Data were collected using Microsoft Excel 2010^®^ (Microsoft, Redmond, USA). Further statistics were performed with SPSS 24.0 (IBM, Armonk, USA). Quantitative parameters (e.g., patients age, days) are given as median and range. Qualitative parameters (e.g., tumor stage) are presented as frequencies. Binary logistic regression was performed to determine the influence of multiple factors on pCR. Possible influencing factors were age, study participation, tumor biology, genetic mutation (BRCA), recurrent cancer, discontinuation of chemotherapy (due to side effects), days between diagnosis and begin of chemotherapy as well as days between end of chemotherapy and operation. All procedures performed in the study involving human participants were in accordance with the ethical standards of the institutional and national research committee and with the 1964 Helsinki Declaration and its later amendments or comparable ethical standards. Informed consent was obtained from every individual participant included in the study.

## Results

Between 2015 and 2018, a total of 205 patients received NACT due to newly diagnosed breast cancer at the Saarland University Medical Center. Twenty-two patients had to be excluded because of insufficient data so that the data of 183 patients were analyzed. The patients median age was 54 years (22–78). The median time between diagnosis and begin of NACT was 28 days (14–91). Between the end of NACT and operation, median time was 28 days (9–57).

Tumor stage and biology, as well as histological subtype and grading are illustrated in Table 1. Twenty-eight patients (15%) had multicentricity. BRCA mutation (9 patients, 5%) and recurrent cancers (7 patients, 4%) were rare. Sixty-two patients (34%) received study therapies: GAIN II (12%), Gepar Octo (10%), GeparX (9%), others (5%). Patients participating in clinical studies had a higher percentage of triple negative tumors (42%) compared to patients without study treatment (26%). Her2 positive tumors are less frequent in the study group (34%), compared to standard therapy regimen (39%). Furthermore, Grading and Ki67 values are higher in patients participating in clinical trials (Grading G3 67% vs. 53%, Ki67 50% vs. 40%). All patients received an Anthracycline and Taxane-based chemotherapy. Fifty-four patients of the Her2 positive group (79%) received chemotherapy in combination with Trastuzumab and Pertuzumab, 21% received chemotherapy in combination with Trastuzumab alone. All patients with triple-negative carcinoma additionally received carboplatin. Thirty-four patients (19%) discontinued NACT. Twenty patients (11%) skipped only the last or the last two chemotherapy doses, whereas 14 patients (8%) received three or more than three doses less. The reasons for a premature discontinuation of chemotherapy were equally distributed in both groups; the major part (16%) due to side effects (polyneuropathy 7%, changes in laboratory values as increased liver values or cytopenia 6%, others 3%). Four patients (2%) discontinued NACT because of tumor progression.

**Table 1:** Tumor characteristics

TNM-stage	Absolute frequencies (*n*)	Cumulative frequencies (%)
*Staging*		
ypT		
0	89	48.6
1	56	30.6
2	16	8.7
3	7	3.8
4	5	2.7
X	10	5.6
ypN		
0	56	30.6
1	26	14.2
2	15	8.2
3	3	1.6
Pre-therapeutic		
pN0 (sentinal node)	83	45.4
pN+	100	54.6

Tumor biology	Absolute frequencies (*n*)	Cumulative frequencies (%)
*Tumor biology*		
NST (invasive carcinoma of no special type)	173	94.5
Others	10	5.5

Histological subtype	Absolute frequencies (*n*)	Cumulative frequencies (%)
*Histology*		
Luminal A		
HR+, Her2 neg, Ki67 ≤ 15%	8	4.4
Luminal B		
HR+, Her2 neg, Ki67 > 15%	50	27.3
Her2 positive, HR positive	48	26.2
Her2 positive, HR negative	20	11.0
Triple negative	57	31.1

Grading	Absolute frequencies (*n*)	Cumulative frequencies (%)
*Grading*		
G1	2	1.1
G2	74	40.4
G3	105	57.4
X	2	1.1

*HR*  hormone receptor

Most patients received breast-conserving therapy (63%), followed by mastectomy (22%) and oncoplastic surgery (15%). Implants or expander was used for 24 patients (13%). The median time of hospitalization during the operation was 4 days (1–22). Patients with breast-conserving therapy had a median time of hospitalization of 3 days (1–14), patients undergoing mastectomy 6 days (2–17) and patients with oncoplastic surgery 7 days (2–22).

A total of 86 patients (47%) had pCR. The patients age, genetic mutation, recurrent cancer, or discontinuation of chemotherapy (due to side effects) did not influence pCR. Likewise, time between diagnosis and onset of NACT, or time between end of NACT and surgery, had no influence on pCR. Patients participating in clinical trials for NACT, higher tumor grade, high Ki67 and Her2 positive tumors had increased chances of having pCR. Patients with Luminal B tumors had a lower chance of achieving pCR. No pCR was detected in the Luminal A group. In patients with triple negative tumors, a trend could be observed. They seem to have more often pCR, although not statistically significant. Influences on pCR are shown in Table 2.

**Table 2:** Influences on pCR by binary logistic regression

Parameter	Odds ratio Exp (*B*)	*P*	95% confidence interval
Age	1.022	0.096	0.996–1.048
Time interval Diagnosis-NACT	0.990	0.450	0.965–1.016
Time interval NACT-operation	0.982	0.285	0.949–1.016
Study participation	2.355	**0.009**	1.244–4.458
Genetic mutation	2.203	0.275	0.533–9.103
Recurrent cancers	0.410	0.294	0.077–2.171
Discontinuation of NACT	1.929	0.115	0.852–4.371
Ki67	1.031	** < 0.001**	1.016–1.047
Grading	4.201	** < 0.001**	2.206–7.999
Triple negative	1.493	0.219	0.788–2.827
Her2 positive HR positive	2.053	**0.045**	1.015–4.150
Her2 positive HR negative	4.500	**0.006**	1.526–13.273
Luminal B	0.297	**0.001**	0.144–0.616

Bold values represents statistically significant

## Discussion

This study presents several influences on pCR, with increased chances in patients participating in studies for NACT, having a high Ki67, high grading or Her2 positive tumors. Tumor subtypes like Luminal A and B had decreased chances of pCR.

Age had no influence on pCR in the current study. In contrast, other studies reported that young age is positively associated with pCR [7]. A cutoff value of 40 years was proposed under which the chances of pCR may be higher [17]. However, pCR can also be achieved in elderly patients, especially for Her2 positive patients [8]. So, age alone should not be an indication for NACT.

Neither time between diagnosis and onset of NACT, nor time between the end of chemotherapy and surgery influenced pCR. Consistently, exceeding a presumed cut-off of 4 weeks for onset of NACT or surgery did not influence pCR and disease-free or overall survival independent of histopathological subgroups [18]. However, it was suspected that patients without pCR could benefit from early surgery [18]. At least time intervals up to 8 weeks between NACT and surgery seem not to influence outcomes [19]. In the present study, the median time from diagnosis to NACT and NACT to surgery was about a month with only a minor part exceeding 8 weeks, which complies with German guidelines [20]. This limits the ability to assess the influence of longer time intervals on pCR. However, our results suggest that therapy intervals seem to have a negligible influence on pCR.

More than a third of our patients participated in clinical trials. Study participants for NACT had more than twice as high a chance to achieve pCR. In addition to higher chances for pCR in patients participating in clinical trials, mastectomy rates were lower [21]. However, one must take into account that the patients who are included in clinical studies are mostly a selected collective. On the one hand, this means that there is a disproportionate part of those patients who have a higher probability to achieve pCR due to tumor biology (e.g. high grading and high Ki67). On the other hand, study patients have fewer previous illnesses due to in- and exclusion criteria which can otherwise lead to premature discontinuation of therapy, dose reduction or longer therapy breaks. In the present study, patients receiving NACT in clinical trials had higher rates of Ki67 and higher grading compared to patients receiving standard therapy. This might be the reason for higher pCR rates in the study participants. Furthermore, there was a higher percentage with triple negative tumors in the study group comparing to standard treatment. However, the rate of Her2 positive tumors (which are also associated with higher rates of pCR) was lower in patients participating in clinical trials. Nevertheless, we should encourage our patients to participate in clinical trials. Besides contributing to therapy improvements, they most likely benefit from new therapies. Possible disadvantages of trial participation, such as delaying the onset of NACT or surgery, could be excluded [11, 12].

Only 5% of our patients had a proven genetic mutation (BRCA) for which a superior response to NACT was previously described [10]. Particularly when BRCA positive patients have triple-negative tumors, better outcomes are suspected [22]. We could not prove a significant influence of genetic mutation on pCR.

Likewise, there was no influence of local recurrent cancers on pCR. To our knowledge, this is the first study analyzing a possible influence of local recurrent cancers on the pCR rate. However, no distinction was made whether patients already received prior chemotherapy. In contrast to that, there are analyses describing the association of pCR after NACT on the recurrence of locoregional cancer. An analysis of 10,075 women showed that the local recurrence rate after chemotherapy was higher in patients with non-pCR (9.5% in 67 months) [23].

Discontinuation of chemotherapy due to intolerable side effects did not influence pCR. Patients discontinuing NACT due to disease progress were not included. In general, guidelines recommend the completion of chemotherapy [20]. Our results imply that discontinuation of chemotherapy may not substantially lower chances for pCR. However, it should be considered that this might be due to the small sample size and that most patients only discontinued before the last or the last two chemotherapy doses. Nevertheless, a detrimental effect of early termination of chemotherapy cannot be excluded. Peripheral neurotoxicity was the most common reason why patients had to discontinue chemotherapy (7%). Unfortunately, therapeutic options like pharmacological treatments are limited [24]. Thus, only dose reduction or discontinuation are current options [24]. Nevertheless, premature discontinuation of NACT seems to be uncritical, especially when a good clinical response was already proven. Further studies are needed to confirm our findings.

Ki67 and high grading were associated with high pCR rates (*P* < 0.001). As Ki67 increases by 1, the probability of a pCR increases by 1.031. This is consistent with findings that especially highly aggressive tumors have a good response to chemotherapy [5, 25]. Triple-negative tumors tend to have better pCR rates without reaching statistical significance. Other studies already showed that triple-negative tumors (as aggressive tumors) were associated with higher pCR rates after NACT [5, 7]. Her2 positive tumors showed higher chances for pCR in our study. This has also been demonstrated in previous studies [26, 27]. It could be shown that patients with Her2 positive, hormone receptor (HR) negative tumors showed the highest rates of pCR. With this tumor biology (Her2+, HR−) the chance to achieve pCR was 4.5 times higher than in other tumor subtypes. Von Minckwitz et al. and Harbeck et al. also observed that Her2 positive tumors respond particularly well to NACT when hormone receptor status is negative [5, 28]. In the present study, even tumors with Her2 positive, hormone receptor positive tumors had a twice as high chance to achieve pCR (*P* = 0.045). This effect could not be shown in all previous studies [5]. One reason for this might be the change in Her2 directed therapy, as the addition of pertuzumab to trasuzumab and NACT led to a more frequent pCR rate [29, 30]. In the present study, most patients (79%) received Trastuzumab and Pertuzumab. Only a minor part (21%) received Trastuzumab alone.

The subtype Luminal B was associated with a lower chance for pCR. Available data seem contradictory. pCR seems to appear more likely in young patients with hormone receptor-positive, Her2 negative breast cancer [7]. However, large analyzes of 13,939 women showed that the lowest rate of pCR was achieved for Luminal A, followed by Luminal B subtypes, whereas pCR rates of triple-negative and Her2 positive were comparatively higher [27]. Also, patients with progesterone negativity showed higher pCR rates [9]. Taken together, high Ki67, high grading and Her2 positive tumors are notably associated with higher pCR. No pCR was detected in the Luminal A group. This seems not to be surprisingly, as Luminal A cancers are not appropriate for NACT and normally show the lowest rates of pCR [27]. In this analysis, the four patients with Luminal A cancers had locally advanced tumors and received chemotherapy therefore neoadjuvant.

This study has limitations. It is single centric, and thus clinical pathways may differ to other centers. However, data were collected from a certified tertiary breast care center, ensuring that therapies are state of the art. The sample size is small, but many other studies are based on registers obtained from clinical trials for new therapy regimes [5, 7–9, 18, 21], which implies selection bias due to specific inclusion criteria. Our data more likely represents “real-world-data”.

## Conclusion

Particularly patients with aggressive tumors (high Ki67, high grading, Her2 positive tumors) had better response rates on NACT. These patients should receive chemotherapy in a neoadjuvant setting. Furthermore, patients participating in clinical trials had higher pCR rates after NACT. Besides contributing to therapy improvements, they most likely benefit from new therapies. We should thus encourage our patients to participate in clinical trials.

## Data Availability

The datasets generated during and/or analysed during the current study are available from the corresponding author on reasonable request.
